# Exercise and dietary recommendations to preserve musculoskeletal health during weight loss in adults with obesity: A practical guide

**DOI:** 10.1007/s11154-025-09968-3

**Published:** 2025-05-28

**Authors:** Jakub Mesinovic, Christopher Hurst, Gloria K. W. Leung, Jack R. Ryan, Robin M. Daly, David Scott

**Affiliations:** 1 Institute for Physical Activity and Nutrition (IPAN), School of Exercise and Nutrition Sciences, Deakin University, 221 Burwood HighwayBurwood, Melbourne, VIC 3125 Australia; 2 Department of Medicine, School of Clinical Sciences at Monash Health, Monash University, Clayton, Melbourne, Australia; 3 AGE Research Group, Faculty of Medical Sciences, Translational and Clinical Research Institute, Newcastle University, Newcastle Upon Tyne, UK; 4 NIHR Newcastle Biomedical Research Centre, Newcastle Upon Tyne Hospitals NHS Foundation Trust, Cumbria Northumberland Tyne and Wear NHS Foundation Trust and Faculty of Medical Sciences Newcastle University, Newcastle Upon Tyne, UK; 5 Institute for Health Transformation, Global Centre for Preventive Health and Nutrition, School of Health and Social Development, Faculty of Health, Deakin University, Geelong, Australia

**Keywords:** Exercise, Diet, Evidence-based recommendations, Obesity, Weight loss

## Abstract

Obesity adversely impacts musculoskeletal health, contributing to functional limitations and an increased risk for falls, fractures and disability. Weight loss can mitigate these effects, but strategies that neglect to incorporate evidence-based dietary and/or exercise approaches can exacerbate musculoskeletal and functional declines. Sustainable weight loss requires a personalised approach that prioritises adequate protein intake and essential nutrients to preserve musculoskeletal health. To enhance adherence and long-term success, dietary strategies should be practical, nutritionally balanced and cost-effective. Similarly, exercise programmes should be individually tailored and progressive, with resistance training central to any program prescribed in the context of weight loss, due to its critical role in maintaining muscle and bone mass and strength. When prescribing weight loss strategies involving lifestyle behaviour changes, clinicians must consider their patient’s readiness to change. We have used the transtheoretical model of change framework as an example to identify a patient’s level of readiness and provided associated motivational interviewing-based strategies to enhance adherence and engagement. This review outlines evidence-based, practical diet and exercise recommendations and behavioural strategies that can facilitate effective and sustainable weight loss, which is particularly important for at-risk populations such as older adults.

## Introduction

Obesity is a major global public health concern with a rising prevalence (43.6 to 57.9% from 1985 to 2014) and significant economic and disease burden (~ 2.19% of global gross domestic product) [1–3]. While widely recognised for its detrimental effects on metabolic and cardiovascular health [4, 5], obesity can also significantly impair musculoskeletal health by worsening muscle composition [6] and performance [7] and increasing the risk of falls [8, 9], fractures [10, 11] and physical disability [12, 13]. Although clinically significant weight loss (≥ 5%) results in significant improvements in cardiometabolic health [14], it can adversely affect musculoskeletal health [15–17]. This review highlights new research on the relationship between obesity and musculoskeletal health, examines the effects of various weight loss approaches on musculoskeletal outcomes, and provides practical exercise and dietary guidance to preserve musculoskeletal health during weight loss.

## Obesity and musculoskeletal health

Obesity has a complex relationship with musculoskeletal health. Higher mechanical loads due to greater body mass can stimulate muscle hypertrophy and bone remodelling, increasing absolute muscle and bone mass and strength [18–21]. Consequently, older adults with obesity generally have ~10–20% higher lean body mass and ~10% higher bone mineral density (BMD) compared to counterparts without obesity [22–24]. However, relative muscle strength (i.e., strength normalised to body size) and physical performance (a multifaceted concept encompassing physical function, balance and the central and peripheral nervous systems) are generally poorer in individuals with obesity [21, 25]. Poor relative strength and physical performance have been associated with 20–70% increased risk of disability, falls and fractures in older adults [26–28].

Myosteatosis, defined as the accumulation of adipose tissue/lipids within and around skeletal muscle (intermuscular/intramuscular adipose tissue [IMAT]), increases in obesity and is associated with poor musculoskeletal health outcomes [29]. Mechanisms driving myosteatosis include obesity-induced low-grade inflammation, mitochondrial dysfunction (e.g. impaired fatty acid oxidation) and disrupted metabolic processes linked to insulin resistance, which further accelerate musculoskeletal declines [30–32]. High IMAT contributes to poor physical performance, independent of muscle mass [33–36]. It is also a stronger predictor of mobility than muscle mass [33] and independently associated with increased fall and fracture risk in older adults [37–39]. Data from the UK Biobank involving 40,178 adults (mean age: 64 years) showed that high IMAT is associated with a 50% increase in mortality risk, independent of confounders including low handgrip strength [40]. Notably, having high IMAT in addition to low muscle volume further increases mortality risk (twofold *vs* normal IMAT and muscle volume), and having both high IMAT and low muscle volume in addition to poor physical function is associated with the highest mortality risk (five–sixfold increase *vs* normal IMAT and muscle volume and function) [40]. Collectively, there is consistent evidence that higher body weight and IMAT, alongside lower relative muscle strength and physical performance, lead to a vicious cycle that perpetuates muscle deconditioning and fat accumulation. This cycle exacerbates physical performance declines and can lead to bone loss and an increased risk for osteoporosis and low trauma fragility fractures [41].

## Sarcopenic obesity

A concerning intersection of obesity and poor musculoskeletal health is sarcopenic obesity, the co-existence of obesity and low muscle mass and function (i.e., sarcopenia) [42]. This condition is associated with increased risk for falls (30%), fractures (29%), cardiovascular disease (6–47%) and mortality (twofold [10-year risk]) [27, 43–45]. In some studies, it has been linked to worse clinical outcomes than obesity or sarcopenia alone [27, 46, 47]. Despite these risks, many individuals with sarcopenic obesity remain undiagnosed, in part due to the lack of consensus on diagnostic algorithms (until the recent ESPEN and EASO Consensus Statement) [42]), but potentially also due to the assumption that obesity is accompanied by elevated muscle mass [18–21]. Sarcopenic obesity prevalence increases with age, reaching up to 48.0% in women and 27.5% in men aged > 80 years [48, 49]. For these individuals, weight loss- a common recommendation for managing obesity [50]- poses a unique challenge. As mentioned, while weight loss may mitigate many metabolic complications of obesity [4, 5], it often leads to losses in both muscle and bone mass when not combined with appropriate exercise and/or nutritional interventions [15–17]. Without adequate screening and musculoskeletal-preserving strategies, older adults with and without sarcopenic obesity may face an even greater risk of adverse outcomes following weight loss.

## Weight loss and musculoskeletal health

Weight loss is typically recommended for those who present to primary care with risk factors for metabolic diseases, such as hypertension or pre-diabetes, if they fall in the overweight or obese category (typically assessed by body mass index [BMI] or waist circumference in clinical settings) [51]. Physiologically, weight loss occurs when energy expenditure outweighs energy intake, which can be achieved via two main behavioural pathways – reducing food (calorie/energy) intake (i.e. energy restriction; ER) and/or increasing energy expenditure (i.e. physical activity; noting that basal metabolic rate and thermic effect of food are difficult to change via lifestyle/behavioural modifications) [52]. ER is a common approach for weight loss, where an individual reduces their daily energy intake below the level required to maintain their current body weight [53].

While ER is effective for fat loss, it also leads to losses in muscle and bone mass and strength [15, 17, 54–57]. Between 10–58% of the weight lost through ER can be comprised of fat-free mass [56, 58], which includes skeletal muscle, whereas decreases in muscle strength are more modest (~3–10%) [55, 59–61]. Losses in BMD are generally around 2% for every 10% decrease in body weight [62]. ER-related muscle and bone mass and strength declines are likely related to several factors, including 1) decreased mechanical loading during daily activities [63–65]; 2) limited availability of essential amino acids [66] and/or insufficient macro- and micronutrient intake [67]; 3) hormonal changes including decreased insulin and insulin-like growth factor-1 [IGF-1] levels alongside elevated cortisol [68–70]; and 4) impaired muscle protein synthesis (MPS) [71]. Although ER decreases absolute muscle mass and strength [17, 72], it improves relative muscle mass and strength, as the proportional decrease in body and fat mass often exceeds the loss of muscle mass and strength during weight loss.

Despite the potential negative consequence of ER-related weight loss on muscle and bone outcomes, it can have beneficial effects on myosteatosis and physical performance. Weight loss decreases IMAT (~20% decrease in IMAT per ~10% reduction in body mass) [73–76], which can enhance physical performance via improved insulin action [76, 77] and muscle contractility (i.e. less fat infiltration improves muscle fibre architecture and force transmission) [78]. Physical performance improvements following weight loss may also occur for several reasons beyond decreases in IMAT, including reductions in the mechanical load on joints and muscles, enhancement of cardiovascular and respiratory function and improved metabolic efficiency [21, 79, 80].

While clinically significant weight loss (≥ 5%) enhances muscle composition and physical performance [17, 73], it can also accelerate muscle and bone loss, increasing the risk of falls and fractures in older adults [81–83]. To mitigate these risks, weight loss strategies should incorporate tailored, multi-component interventions designed to improve both cardiovascular and musculoskeletal health.

## Weight loss approaches

Broadly speaking, there are three main approaches to induce an energy deficit to achieve weight loss – behavioural interventions (e.g. diet and exercise), surgical interventions (i.e. bariatric surgery), and pharmacological interventions, with the latter two ideally implemented in combination with behavioural interventions.

Indications for each of these treatment options are outlined in the 2020 ‘ESPEN guidelines for obesity treatment’ [50], which summarises current international recommendations for weight management in adults including the 2013 US guidelines ‘Management of Overweight and Obesity in Adults’ [51] and the 2014 UK guidelines ‘Obesity: identification, assessment and management’ [84]. In brief, recommended treatment options depend on BMI class, comorbidities and previous weight loss attempts. The 2013 US guidelines [51] and the Australian Obesity Management Algorithm recently published by the Australian Diabetes Society [85] include detailed algorithms to guide health professionals in selecting treatment options. A description of the above guidelines and treatment options is not the focus of the current review; however, we have drawn from these in our recommendations for behavioural interventions for weight loss that preserve musculoskeletal health.

### Behavioural interventions – dietary strategies

A food-based ER diet is a key component of behavioural interventions for weight loss in adults with overweight (BMI 25–30 kg/m^2^) and comorbidities; or with grade I obesity (BMI 30–35 kg/m^2^) [50]. Weight loss of 5 to 10% within 6 months is the suggested initial goal, as this is considered the minimum required for clinically significant metabolic health improvements [51]. This can be achieved through reducing or modifying food intake, to induce an energy deficit of 500 to 1000 kcal (~ 20% energy requirements) per day, promoting a gradual weight loss of 0.5 to 1.0 kg per week [50]. Over the past century, numerous weight loss diet strategies have been developed, with varying levels of scientific evaluation and evidence supporting their effectiveness. Those that have been studied scientifically can be broadly categorised into three groups: dietary strategies based on i) manipulating macronutrient content (e.g. low-carbohydrate or low-fat), ii) restricting specific foods or food groups (e.g. Paleolithic or Mediterranean diet), and iii) manipulating timing of food intake (e.g. intermittent fasting [IF]) [86].

Among these strategies, altering macronutrient composition and restricting specific foods and food groups have been the most widely studied for their effects on weight loss. A network meta-analysis (NMA) of 121 randomised control trials (RCTs) found that at six months, diets such as low-carbohydrate and low-fat led to similar reductions in body mass (4.63 vs. 4.37 kg) compared with a usual diet (no ER) [87]. Comparable weight loss was also observed with popular named diets such as the Mediterranean and Paleolithic diets (2.87 vs. 5.32 kg, respectively) compared with usual diet [87]. Of note, by 12 months, all of the 14 named diets included in this NMA except for the Paleolithic diet had a treatment effect that was approximately 1.5 kg smaller, highlighting the challenges associated with achieving long-term reductions in weight [87]. These findings also illustrate that while dietary strategies that manipulate or restrict macronutrients or foods vary in composition, a key determinant of weight loss across all approaches is the creation of an energy deficit, and long-term adherence is essential for sustained benefits.

In recent years, IF has emerged as a potentially effective dietary strategy for weight loss. The basis of IF regimes is manipulation of meal timing; with common approaches including alternate-day fasting and the 5:2 diet, which prescribes 500 to 660 kcal on fasting days. Using the 5:2 diet as an example, as it prescribes two fasting days during the week and allows for habitual dietary intake for the other five, some studies have shown that adherence to IF may be easier compared to other dietary strategies, as patients only have to think about ‘dieting’ on two days of the week [88, 89]. Recent systematic reviews of RCTs have reported weight loss achieved by alternate day fasting and 5:2 diet is comparable to daily ER [90, 91]. Time-restricted eating (TRE) is another common IF regime whereby feeding is restricted to a particular window of the day (ranging from 4 to 12 hours) [92]. It is currently unclear whether TRE alone can lead to clinically significant weight loss. A recent meta-analysis of 7 RCTs suggests TRE (without ER) leads to greater weight loss (mean difference: 2%) compared to no time restriction or an extended eating window, however, this seemed to be driven by lean mass losses rather than fat mass losses [92]. Experimental studies incorporating TRE with ER have reported conflicting findings [93, 94], with any additional weight loss driven by unintentional reductions in energy intake rather than a unique metabolic advantage [93].

Current obesity management guidelines do not favour one dietary strategy over another for weight loss [50]. As mentioned, effectiveness will vary between individuals, depending on the ease of adherence in the context of their lifestyle [95]. Despite their efficacy in promoting weight loss, all dietary strategies have the potential to induce muscle and bone loss if weight loss is of a sufficient magnitude (and not combined with appropriate exercise), which can have significant implications for long-term health, particularly in older adults. For instance, a 2-year RCT of 307 adults with obesity comparing low-carbohydrate to low-fat diets reported that both dietary approaches resulted in a 5% reduction in lean mass and a 1.5% reduction in BMD at the total hip and lumbar spine [96]. As previously mentioned, IF can also lead to lean mass losses [92], but these do not seem to be greater compared with continuous ER in short-term studies (< 24 weeks) [97]. Very few RCTs have explored the effects of IF on bone health. A recent review found that only 2/8 RCTs assessing bone changes following IF interventions were of a duration sufficient to observe real physiological changes in BMD (> 6 months), and neither study assessed clinically relevant sites such as the total hip and lumbar spine [98]. Nevertheless, the extent of lean and bone mass loss following weight loss depends on several factors, including overall diet quality, micro/macronutrient intake (i.e., protein, calcium and vitamin D etc.), and whether musculoskeletal-preserving strategies, such as resistance training (RT), are prescribed concurrently with dietary interventions. Diet and exercise strategies for preserving musculoskeletal health during weight loss are discussed later in this review.

#### Very-low-calorie and low-calorie diets

ESPEN guidelines recommend very-low-calorie diets (VLCD) and low-calorie diets (LCD) as potential weight loss dietary strategies for individuals with a BMI 30–40 kg/m^2^, to achieve the targeted weight loss of 10 to 20% [50]. These dietary interventions typically rely on commercially available liquid formulas and meal replacements to meet the prescribed daily energy intake of < 800 kcal for VLCDs or 800–1200 kcal for LCDs [50, 99]. Without the incorporation of such medically formulated replacements and relying on food exclusively, it can be difficult to achieve protein requirements within such a restricted-energy quota. Given these restrictions, there is concern regarding the impact of VLCD/LCD on musculoskeletal health. A systematic review reported a positive association between increasing ER (from LCD to VLCD) and fat-free mass loss, suggesting that more restrictive diets may have adverse effects [100]. In contrast, a meta-analysis of 7 RCTs reported that LCD/VLCD does not lead to greater fat-free mass reduction compared to moderate ER (500 to 750 kcal deficit per day) [101]. Notably, these interventions were only prescribed for short durations (5–12 weeks), but greater reductions in resting metabolic rate were observed, which may impact long-term weight loss and maintenance. Nonetheless, due to its risk of causing nutrient deficiencies, safe implementation of LCD and VLCD requires monitoring by a health professional (e.g. GP or dietitian), and they should only be undertaken for a maximum period of 12 weeks [50].

Potential musculoskeletal risks associated with severe ER were highlighted in a 12-month RCT involving 101 postmenopausal women (mean age = 58.0 years) with obesity [102]. This study found that a VLCD using complete meal replacements and providing 1 g/kg/day of protein resulted in nearly double the weight loss (17.3 vs. 8.8%) compared to a food-based moderate ER diet [102]. Despite the greater weight loss, there were no additional adverse effects on relative whole-body lean mass or handgrip strength in the VLCD group [102]. However, the VLCD group experienced disproportionately greater losses in BMD, with a 2.1-fold greater reduction in total hip BMD (0.032 vs 0.015 g/cm^2^) [102]. Notably, despite meeting protein intake recommendations for older adults and providing adequate dietary calcium and vitamin D, the VLCD group still experienced greater bone loss, highlighting the need for caution when prescribing severe ER, particularly in postmenopausal women or individuals with or at risk of osteopenia and osteoporosis.

#### Special considerations for older adults

Age-related declines in musculoskeletal health necessitate different considerations for weight loss in older adults. ESPEN guidelines suggest that for older adults in the overweight category (BMI ≥ 25 and ≤ 30 kg/m^2^), weight loss via ER is not recommended; implementation of dietary strategies should be aimed at weight maintenance and metabolic improvements [103]. For older adults with obesity (BMI > 30 kg/m^2^), weight loss via ER may be considered, but potential benefits must be weighed against potential adverse effects, such as impacts on muscle health and strength and physical functioning on a case-by-case basis [103]. Where weight loss is deemed appropriate, ER should not exceed a 500 kcal/day deficit and the dietary strategy should be food-based. Whilst there is no clear guidance on protein intake required to eliminate the risk of sarcopenia and osteoporosis, the ESPEN guidelines emphasise that the dietary strategy should provide at least 1 - 1.2 g/kg/d of protein (recommendation for the general older adult population) [103]. Further, due to their increased risk of malnutrition, a VLCD or LCD are not recommended for older adults [99, 103].

### Behavioural interventions – exercise

Aerobic training (AT) is a structured form of physical activity characterised by continuous, rhythmic movements involving large muscle groups, such as running, cycling, and swimming. It is effective for improving cardiometabolic health, reducing visceral and subcutaneous fat (at higher exercise doses) and improving cardiorespiratory fitness [104, 105]. A NMA of 45 RCTs showed that relative to control, moderate intensity or vigorous AT decreases body mass (−0.65 to −0.75 kg), BMI (−0.94 to −1.76 kg/m^2^) and waist circumference (−2.03 to −2.31 cm) [106]. While AT is effective for improving aerobic fitness (a strong predictor of mortality) and can lead to small gains in muscle mass [107], it provides very limited benefits for bone mass, as it generally lacks the necessary mechanical loading (moderate to high and/or novel impact forces) required to elicit strains on bone to trigger adaptations [108]. Nevertheless, AT and other forms of exercise are not recommended as standalone approaches to achieve clinically significant weight loss but should be included adjunct to dietary interventions or other weight loss therapies.

### Endoscopic bariatric therapies

Bariatric surgery (BS) is an effective intervention for rapid and sustained weight loss in individuals with obesity (BMI > 35 kg/m^2^) [109] and related comorbidities that consistently demonstrates greater weight loss than behavioural (i.e. dietary) interventions (~ 20–70% depending on the surgery type) [110–112]. By surgically altering the digestive system, such as through the Roux-en-Y gastric bypass (RYGB), adjustable gastric banding, or sleeve gastrectomy, BS significantly reduces food intake and/or absorption in the long term, thereby increasing the chances of weight loss being maintained [113]. However, a key concern following BS due to the rapid and large amount of weight loss is the impact on musculoskeletal health.

BS leads to a marked reduction in both fat and lean mass, with losses being most pronounced in the first year following surgery [114–117]. Muscle mass losses following BS can account for anywhere between 18–44% of total weight lost [116–118], which is of particular concern as it contributes to BS-related bone loss [119, 120]. In a prospective study of 48 adults with obesity (BMI: 44 ± 8 kg/m^2^), RYGB led to a clinically significant 8% reduction in total hip and femoral neck BMD after 12 months [119, 121]. BS also decreases absolute muscle mass and strength but improves relative strength and physical performance [122]. The same study showed that at 12 months post-surgery, appendicular lean mass was reduced by 16% and absolute hand grip strength declined by 9%, whereas relative hand grip strength (normalised to BMI) increased by 32%, gait speed increased by 13% and 400-m walk time decreased (improved) by 14% [122]. However, these improvements in relative strength and physical performance do not appear to confer protection against fragility fractures with relative risk for pelvis, hip and femur fracture increasing nearly threefold following BS [123].

Non-surgical interventions, such as intragastric balloons, which involve endoscopic insertion of a silicon fluid-filled balloon into the stomach to promote early satiety, have also been shown to result in lean mass losses [124, 125]. Weight loss from this intervention is generally more modest (~ 9.5%) [124] than BS, but further studies are required to understand whether musculoskeletal and physical function changes are similar. It is also unclear whether weight loss from this intervention is associated with an increased risk for falls or fractures.

In summary, BS offers an effective, long-term, solution for weight loss, particularly for those struggling with severe obesity. One major drawback of BS is the irreversibility of most procedures, which can result in gastrointestinal issues, increased difficulty in meeting nutrient requirements through food, and the exacerbation of nutrition-related problems. These challenges are particularly concerning for older adults, especially when nutritional demands rise due to illness or incidents such as infection, viral illnesses, or trauma. Pharmacological therapies such as new-generation incretin mimetics have emerged as another viable method for facilitating weight loss in individuals with obesity or obesity-related complications.

### Pharmacotherapy – incretin mimetics

Incretin mimetics, such as glucagon-like peptide-1 (GLP-1) receptor agonists, were initially developed for type 2 diabetes management but are increasingly used for treating obesity [126]. These agents (in conjunction with lifestyle interventions) are a treatment option for individuals with a BMI > 30 kg/m^2^ or those with a BMI of 27–30 kg/m^2^ who have obesity-related complications [127].

New-generation incretin mimetics like Semaglutide, Tirzepatide, and Retatrutide achieve greater weight loss than older-generation incretin mimetics, largely due to their prolonged half-lives, improved tolerability or additional mechanisms of action (e.g., Tirzepatide is a dual GLP-1 and gastric inhibitory polypeptide receptor [GIP] receptor agonist while Retatrutide is tri-agonist targeting GLP-1, GIP and glucagon receptors) [128]. RCTs demonstrate significant weight loss of 16% (Semaglutide; 68 weeks) [129], 21% (Tirzepatide; 72 weeks) [130] and 24% (Retatrutide; 48 weeks) [131]. However, these interventions are also associated with substantial lean mass losses comprising of ~ 25% and ~ 39% of total weight loss for Tirzepatide and Semaglutide, respectively [130, 132]. It is unclear whether Retatrutide leads to similar body composition changes. Lean mass losses seen with GLP-1 agonists are comparable to those induced by bariatric surgery or dietary interventions [133]. Differences in percentage lean mass losses caused by Semaglutide and Tirzepatide in large trials such as STEP-1 [132] and SURMOUNT-1 [130] could be attributed to medication differences or adherence to lifestyle counselling provided in the trials (e.g., ≥ 150 min/week physical activity and hypocaloric diets with ~ 20% protein intake); lean mass data has not been reported in head-to-head trials [134, 135]. Similarly, evidence on the effects of these interventions on physical function is limited. Most studies report improvements in self-reported measures, such as SF-36 physical function scores [130–132], but objective outcomes are rarely assessed [136].

Older generation incretin memetics (e.g., Liraglutide) have been linked with improvements in BMD, but these drugs generally do not lead to the magnitude of weight loss observed in response to newer drugs, and they were mainly tested and prescribed as a treatment for type 2 diabetes [137]. The pathophysiology of type 2 diabetes increases fracture risk due to impairments in bone material properties and disease-related complications [138, 139], so GLP-1 agonists may mitigate some of these risks via glucose-lowering effects. A meta-analysis of 44 RCTs in adults living with T2DM reported a 23% reduction in fracture risk in adults prescribed GLP-1 receptor agonists (analysis included both older and newer agonists) with beneficial effects seen in long-term studies (> 78 weeks) and among Liraglutide users [140]. However, sub-analyses for Semaglutide and Tirzepatide were non-significant, likely due to lack of statistical power [140]. For newer incretin mimetics, evidence remains limited, though a recent RCT in adults with increased fracture risk (T-score < −1 at the hip or lumbar spine and/or low-energy fracture within 3 years of recruitment) noted decreases in BMD at the total hip and lumbar spine and elevated bone resorption (increased C-terminal telopeptide of type 1 collagen levels) without changes in bone formation (total procollagen type 1 N-terminal propeptide levels) after 12 months of Semaglutide [141]. Further studies are required to determine how new-generation incretin mimetics influence bone heath and fracture risk in individuals without type 2 diabetes.

Finally, it has been suggested there is insufficient data to support the notion that incretin-based therapies and their associated muscle loss increase the risk of frailty, falls and/or sarcopenia [142]. However, additional research is needed to assess whether marked weight loss induced by these agents affects muscle mass, strength and function in high-risk populations (e.g. women during menopause and older adults with and without sarcopenic obesity), using objective physical function assessments and gold-standard measurement methods of muscle mass such as magnetic resonance imaging, computed tomography and the D3-creatine dilution method [143].

## Diet and exercise strategies for preserving musculoskeletal health during weight loss

Maintaining musculoskeletal health during weight loss requires targeted strategies that mitigate the adverse effects of ER on musculoskeletal health while maximising the benefits (i.e., fat loss and cardiometabolic health improvements). A combination of evidence-based dietary and exercise interventions can be used to achieve this. This section highlights the most effective strategies and provide practical recommendations for healthcare providers. Together, these strategies aim to support successful and sustainable weight loss whilst optimising musculoskeletal health and function.

### Dietary strategies-protein, calcium, vitamin D

Protein, an essential macronutrient, is key to maintaining muscle mass and other metabolic functions. Protein-rich meals stimulate MPS, primarily driven by essential amino acids like leucine (at least 2–3 g is required to trigger MPS), while insulin suppresses muscle protein breakdown [77, 144]. However, aging appears to reduce the anabolic response to protein intake and diminishes the anticatabolic effect of insulin [145, 146], meaning that a higher dose of protein is required to achieve the same robust anabolic response observed in younger adults. Most guidelines suggest a recommended daily intake (RDI) [145, 146] of ~ 0.8 g/kg/day in adults and up to 1 g/kg/day in older adults [145], but there is emerging evidence that protein intakes should be increased to ~ 1.2–1.6 g/kg/day in older adults [56, 147, 148]. Systematic reviews of RCT suggest that protein intakes > 1.0–1.3 g/kg/day promote fat loss [56, 147–150] and can attenuate ER-related losses in lean (muscle) mass [56, 147, 151–153]. Increased protein intake during ER enhances fat mass loss by promoting satiety, increasing thermogenesis and supporting the preservation of lean mass [154]. Several studies suggest an upper limit to the benefits of increasing protein intake, with intakes above 1.6 g/kg providing little additional benefit [155]. With respect to meal frequency and timing, evidence suggests between 20–30 g of protein intake per meal consumed 3–4 times per day leads to optimal MPS and appetite control [154, 156, 157], however, similar body composition and physical function changes are observed when protein is consumed during shortened feeding windows (e.g. during TRE) in adults with obesity undertaking ER [94, 158–160]. Therefore, while evenly distributing protein intake may optimise MPS and appetite control, total daily protein intake appears to be the primary determinant of body composition and physical function changes during ER.

It was previously thought that excess protein intake has adverse effects on BMD [161]. However, newer research has shown protein supplementation has no adverse effects on BMD with a meta-analysis of 16 RCTs and 20 prospective cohort studies showing that higher protein intake has no adverse effects on BMD at most skeletal sites, and a potential protective effect on lumbar spine BMD (compared with lower protein intake) [162]. Protein intake also seems to have beneficial effects on bone health during weight loss. In a 12-month RCT in 47 postmenopausal women undertaking ER (500–600 kcal deficit/day), a high-protein diet (30% of total calories) attenuated BMD loss at the ultradistal radius, lumbar spine and total hip, compared with a normal-protein diet (18% of total calories) [163]. While protein intake is critical in preserving musculoskeletal health during ER, consuming adequate amounts of other key micronutrients is also essential.

Calcium and vitamin D are two of the most extensively studied micronutrients for optimising musculoskeletal health. Both are important for maintaining bone health and lead to a small reduction in fracture risk [164], with some evidence suggesting vitamin D supplementation has small benefits on muscle strength in individuals with 25-hydroxyvitamin D levels below < 25 or 30 nmol/L [165, 166]. However, recent meta-analyses of RCTs show that vitamin D and calcium supplementation have no effect, or potential adverse effects, on physical performance [167, 168]. Of the few RCTs that have explored the effect of these micronutrients on musculoskeletal outcomes (e.g. muscle mass, strength and performance and BMD) during ER, most report small or negligible benefits of supplementation [169–173]. Evidence highlights potential roles for other nutrients, including vitamin K1, magnesium and vegetable-derived nitrate, in maintaining musculoskeletal health and preventing falls and fractures [174, 175], but further research is required to better understand how altering intake of these nutrients influences ER-related changes in musculoskeletal outcomes.

#### Key elements of dietary strategies for weight loss:

Based on the above review of currently available evidence, below are key elements that should be considered when selecting a dietary strategy for weight loss in adults and older adults.

Adults

- BMI 25 to 35 kg/m^2^: food-based diet with a calorie deficit of 500–1000 kcal per day (equivalent to a reduction of ~ 20% of energy requirements)
- Protein intake of 1.2 to 1.6 g/kg body weight/day, where exercise is incorporated, to minimise loss of lean body mass. Current evidence suggests minimal additional benefits beyond 1.6 g/kg/day.
- When selecting a food-based dietary strategy, consider comorbidities, as well as their perceived ease to implement and follow for a period of ~ 6 months.
- BMI 30 to 40 kg/m^2^: LCD and VLCD may be considered but should not be undertaken for more than 12 weeks. These dietary strategies may be particularly helpful for individuals with BMI of 35 to 40 kg/m^2^ to ‘kickstart’ weight loss.
- Assess dietary calcium intake and serum vitamin D levels; supplement to national recommended intake levels if required.

Older adults

- BMI 25 to 30 kg/m^2^: dietary strategy should be aimed at weight (and muscle) maintenance and improving metabolic risk factors, rather than weight loss.
- BMI > 30 kg/m^2^: Food-based diet with a maximum calorie deficit of 500 kcal/day can be considered, after potential risks to functioning and other comorbidities have been examined.
- Protein intake of ≥ 1.2 g/kg/day when following ER-diet for weight loss, even in the absence of exercise, to attenuate muscle loss.
- Protein intake of ≥ 1.6 g/kg/day when combining ER diet and exercise for weight loss.
- LCD and VLCD are not recommended.
- Assess dietary calcium intake and serum vitamin D levels; supplement to national recommended intake levels if required.

### Multicomponent Exercise Interventions

Exercise (resistance-based training) attenuates weight loss-related declines in musculoskeletal health, with the extent of protection being largely dependent on the mode and prescription factors (i.e., dose) [15, 16]. Progressive RT is the most effective mode for maintaining/improving muscle size and strength, but as with any exercise mode, not all exercise prescriptions are equally effective for deriving optimal gains [176]. When delivered appropriately, the *exercise dose* drives the stimulus for adaptation [177]. As such, the structure of the exercise dose needs to be effectively manipulated for each individual to induce the desired adaptations.

A wide range of RT programmes can induce positive responses provided they follow the fundamental principles of specificity, overload, and progression [178, 179]. Specificity refers to the concept that training adaptations are specific to the exercise stimulus applied. As such, exercises should be prescribed to specifically target the goals of the individual. Adhering to the principles of overload (a greater than habitual stress or load on the body) and progression (a gradual and systematic increase in stress placed on the body) is necessary to induce continual training adaptation over time [180].

As previously described, in high doses, AT can contribute to weight loss, but there are concerns around muscle and bone loss when combined with ER. For instance, a 6-month RCT in 160 older adults (aged > 65 years) with obesity undertaking ER (500–750 kcal/day deficit) found that participants additionally prescribed AT experienced significantly greater losses in lean mass (–5%) compared to those prescribed RT (–2%) or combined AT + RT (–3%) [181]. Total hip BMD declines were also greater in AT (–2.6%) compared with RT (< –1%) and AT + RT (–1.1%) [181]. However, combined RT and AT resulted in greater improvements in a composite physical function score (21%) compared with AT (14%) and RT (14%) alone, although this may be explained by the higher training dose in the combined group [181]. Similar effects of AT and RT on musculoskeletal outcomes during ER have been reported elsewhere [57, 182–184] and in the context of other weight loss approaches such as BS [125, 185, 186] and incretin-mimetics [187, 188], although RCTs prescribing RT in conjunction with new-generation drugs are lacking. Collectively, these findings reiterate the importance of incorporating *targeted* exercise during weight loss, where RT should ideally be central in any exercise prescription with AT being viewed as a complement to RT to optimise aerobic fitness, rather than an alternative.

## Practical resistance training prescription

Practical RT prescription is a product of several primary programming variables, including 1) training frequency, 2) exercise selection, 3) exercise intensity, 4) exercise volume (total number of sets and repetitions) and 5) rest periods. Manipulating these variables can modify the stimulus and adaptations induced by RT (see [178] for further discussion). To maximise outcomes, exercise practitioners should ensure RT programmes are adapted to the specific needs of each individual, embedding variation within the training programme to keep the stimulus novel.

It is likely that a similar RT prescription to that which would be recommended outside of the weight loss setting (i.e. a total body workout performed 2–3 times per week, targeting 1–2 exercises for each major muscle group in each exercise session) represents a sensible approach to optimising musculoskeletal health and function during weight loss. Ensuring that exercises are performed with the correct form (technique) should be a primary consideration for all programs. Alternating between upper- and lower-body exercises can allow muscles to rest while other muscle groups are trained. An illustrative RT prescription is presented in Table 1.

**Table 1:** Weekly exercise prescription to optimise musculoskeletal health during weight loss

**Resistance Training (2–3 sessions)—Progress to 60 min max per session**	
Exercise selection	6–8 exercises per session e.g Lower-body Leg press/squat Knee extension Leg curl/hamstring march Calf raise Upper-body Chest press/push up Shoulder press Seated Row Pull down/up
Exercise intensity	Moderate – high* RPE 4–5 progressing to 6–8
Exercise volume	3 sets of 6–12 repetitions
Rest periods within sessions	1–3 min
Rest period between sessions	≥ 48 h
**Aerobic activity (3–5 sessions)**	
Exercise selection	e.g. Brisk walking, jogging, cycling, swimming, rowing, elliptical etc
Exercise intensity	RPE 5–8
Exercise volume	30–60 min
**Functional/balance/impact training (generally recommended for older adults; 1–2 days)**	
Exercise selection	e.g. Sit-to-stand, standing tandem balance, singe-leg hops
Exercise intensity	RPE 5–8 or use a similar approach to rate how challenging the activity is (i.e. how difficult it is to maintain balance)
Exercise volume	e.g. 1–3 sets of 6–12 reps for functional exercises Hold balance tasks for 30–60 s 50–100 impacts

*A range of intensities can be effective. Individuals should be encouraged to perform the exercise with a relatively high degree of effort. RPE, rating of perceived exertion

### Resistance training progression

For exercise practitioners, several approaches can be used to ensure progressive overload throughout the programme [179]. Decisions about how and when to progress should be individualised and consider tolerance of the exercise (e.g. whether the increase in training load is achievable for the individual), the specific goals of the RT programme, and enjoyment. Monitoring exercise intensity using ratings of perceived exertion (RPE), where the individual is asked to provide a subjective evaluation of how strenuous the exercise was, can be a useful approach to ensure exercise is performed at an appropriate intensity [189] and determine whether exercise prescriptions are achievable. Some suggestions for how to progress the RT programme can be found in Table 2.

**Table 2:** Strategies for ensuring progressive overload during a resistance training programme

Training frequency	• Increase the number of RT sessions per week
Exercise selection	• Increase the complexity of exercises performed in a training session (i.e., single-joint vs. multi-joint exercises) • Alternating exercises between training sessions can help to ensure variation in the training programme
Exercise volume	• Increase the number of exercises performed in a training session • Increase the number of sets performed per exercise (e.g., 2 sets progressing to 3 sets) • Increase the number of repetitions performed per set
Exercise intensity*	• Increase the intensity (load lifted) of an exercise • A wide range of RT intensities can induce meaningful improvements in strength however, higher intensities are needed to improve maximal strength • Higher speed of movement also contributes to the intensity of exercise and manipulating this can lead to differential adaptation (higher speed of movement = lighter load lifted)

* an inverse relationship exists between volume and intensity, the higher the intensity, the fewer repetitions that are typically performed

### Further considerations for prescribing exercise

Exercise practitioners prescribing or delivering exercise should be mindful of issues such as exercise-induced hypoglycaemia, hypertension (and transient changes in blood pressure during exercise) and joint pain associated with osteoarthritis which are likely to be common in individuals with obesity. However, alternative exercises can be selected if pain impacts the ability to perform a particular exercise. Careful and ongoing monitoring during and post exercise sessions as well as providing individuals with the opportunity to be actively involved in the design of their exercise programme can ensure that these risks are managed effectively.

## Maximising the potential of diet and exercise interventions

Physiologically, evidence suggests numerous dietary approaches to be effective in inducing weight loss while preserving musculoskeletal health. However, as with any behaviour change interventions, the key determinant of success is adherence. Dietary strategies should therefore be tailored to individual’s health needs and social context, to maximise adherence to a minimum period of six months, which is the typical recommended duration to achieve significant weight loss. It is important to note that weight loss dietary strategies should not be viewed as ‘lifelong’ treatments. Once the targeted body weight is achieved, elements of the dietary strategy can be maintained as part of healthy eating, but the dietary strategy no longer needs to be followed strictly, as the overall goal would change to maintaining body weight and overall health.

Similarly, a well-designed exercise programme may not be enough to ensure that beneficial outcomes are fully realised for an individual. Several factors can contribute to the effectiveness of an exercise programme. Firstly, it is important to note that exercise should be prescribed and delivered by those who are qualified and experienced in doing so (e.g., clinical exercise physiologists or others with specialist training in exercise prescription). In the broader context, the perceived value of exercise and how it is delivered within the clinical pathway requires further consideration. All those responsible for care or support of the individual have a role to play in promoting the benefits of exercise in this context. To promote engagement, exercise programme goals need to be developed in conjunction with the exerciser who should be an active participant, as opposed to a passive recipient, in this decision-making process. Exercise practitioners should not lose sight of the importance of making sure exercise is enjoyable and should seek to provide opportunities for social engagement (e.g., group-based activities) wherever possible [190]. Exercise programmes should also be supplemented with educational activities to support ongoing engagement [191]. For example, particularly in those who are less experienced, it is important to make clear that muscle soreness is a normal and expected consequence following RT.

Beginning behaviour change is undoubtedly difficult. In Table 3, we have presented an example of behavioural weight loss strategy, which incorporates the diet and exercise recommendations outlined in this review. Of note, intermittent fasting was used as an example, as it requires comparatively low mental load to implement, which may be a helpful strategy to clients to start with.

**Table 3:**
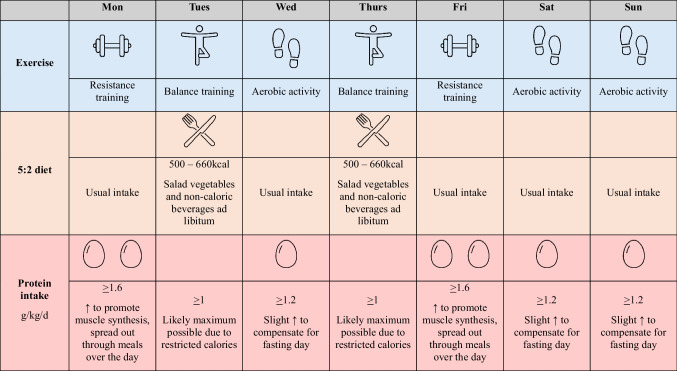
Example weekly weight loss strategy incorporating intermittent fasting (5:2) and exercise

This example is designed for individuals who tolerate exercise well. Prescribers can modify it by incorporating rest or low-intensity days as needed.

## Recognising readiness for change

For lifestyle interventions to be effective, individuals must be at the appropriate stage of readiness to adopt and sustain these changes. Without a genuine commitment to engaging in new behaviours, even the most well-designed interventions may fail to achieve meaningful outcomes.

### Behaviour change

The transtheoretical model of change (TTM) [192] identifies key factors influencing the stages of changes individuals undergo when adopting new behaviours or making lifestyle modifications. The theory contains five stages: precontemplation, contemplation, preparation, action and maintenance (Table 4) [192]. The strength of TTM lies in tailoring interventions to an individual’s stage of readiness to change, which can support sustainable health behaviour improvements. Assessing an individual’s readiness to change and tailoring interventions accordingly can increase adherence and maintenance of behaviour change. Depending on the individual’s stage of readiness, intensity of interventions would vary but typically aim to at least progress clients to the next stage of readiness. TTM has been frequently used by clinicians to support their patients in adopting core behaviour changes that prevent development of chronic diseases, such as healthy eating, increasing exercise and smoking cessation [193]. However, the model’s effectiveness may diminish when addressing several behaviours simultaneously [194]. This decline is due to the increased complexity and cognitive demands placed on individuals, making it harder to maintain progress across multiple behaviour changes. Therefore, when implementing concurrent changes in diet and physical activity, it is best if they alternate in requiring cognitive demands, such as that suggested in the sample strategy (Table 3), where there is only one focused behaviour change each day. RCTs demonstrate that TTM can facilitate weight loss (mean difference: 0.2–2.1 kg) compared with control [195, 196], but it is important to consider that participants in these trials are often already in the ‘action’ stage, having likely surpassed the earlier stages of precontemplation, contemplation and preparation before enrolling the study. This limits the generalisability of the findings to individuals in earlier stages of readiness to change.

While TTM outlines the stages individuals progress through during behaviour change, MI serves as a complementary approach that provides the communication techniques needed to support individuals at each stage, fostering engagement and self-efficacy in the change process. Rollnick and Miller [197] describe MI as a person-centred counselling method aimed at resolving ambivalence and supporting behaviour change. By prioritising the individual's values and goals, MI helps clients make decisions that align with their intrinsic motivations. A systematic review of RCTs (k = 72) evaluating the efficacy of MI in treating lifestyle problems (e.g. smoking) and diseases (e.g. obesity) reported that in 72% of the trials it included, MI outperformed traditional advice and was effective even in short encounters (15 min; 64% of trials showed an effect) but more effective in longer (60 min; 81% of trials showed an effect) or multiple encounters (87% in studies with > 5 encounters) [198]. In the context of weight loss and physical activity specifically, systematic reviews have reported some benefits of incorporating MI, with around one-third of studies reporting that MI led to greater weight loss (up to 5%), muscle strength improvements and reductions in sedentary behaviour [199–201]. An example of MI-based support strategies to support patients in implementing behaviour change for weight management is provided in Table 4.

**Table 4:** Recommended approach and MI-based strategies for each stage of change to support patients in implementing behaviour change for weight management

Stage of Change	Description of patient behaviour	Suggested approach	MI support strategies
Precontemplation	Patients in this stage do not recognise the health consequences associated with being overweight or obese and lack intention to change	• Educate on potential health impacts (e.g., increased risk of chronic disease and disability) and associated impact on quality of life • Provide gentle, non-judgemental advice tailored to their personal goals and values	• **Express empathy** and avoid confrontation • Highlight discrepancies between current behaviours and desired outcomes • **Acknowledge autonomy** to build trust
Contemplation	Patient is aware of benefits of weight loss and is considering behaviour change. They evaluate benefits like better health against perceived sacrifices. Common thoughts include, “I know I need to eat healthier, but I’m not ready yet”	• Support them to weigh the pros and cons of change • Where possible, refer to dietetics and exercise professionals to prepare programs	• **Guide patients through a decisional balance exercise** to evaluate both the benefits of their behaviour and the potential gains of change • Listen for **"change statements"**, such as expressions of concern or intent to change, and reflect them back to the client • **Assess** how long the patient has been contemplating change and explore past attempts to identify barriers • **Foster self-efficacy** by instilling hope and confidence that change is achievable • Be **patient and persistent**, as moving from contemplation to preparation often takes time and multiple discussions
Preparation	Patient has decided to take action. Have started to research ideas such as healthy meal ideas or exercise classes that are available locally. May have made small steps towards change, e.g. cutting out one sugary drink per day or signing up to the gym	• Support to develop a realistic plan for action, with small achievable goals to begin with and a main goal. Collaborate on planning to increase patient ownership of the process • Offer a menu of choices (each geared toward positive change outcomes) and incremental options for change	• **Offer Options & Support**: Present various strategies for change, and discuss available support systems to enhance accountability and success • **Identify Barriers & Solutions**: Explore potential obstacles and develop strategies to overcome them, ensuring the patient feels prepared for challenges • **Strengthen Confidence & Commitment**: Reinforce the patient’s strengths, build confidence, and encourage firm commitment to action, while acknowledging any ongoing ambivalence
Action	Patient is consistently implementing behaviour changes to manage weight	• Monitor progress and review plan to ensure behaviour change is still progressing towards main goal • Celebrate successes • Provide strategies to overcome setbacks or barriers (e.g., meal planning, other life events being prioritised)	• **Support & Reinforce**: Offer ongoing support to help patients stay on track and address any conflicting feelings they may have about the changes • **Acknowledge Effort**: Recognise the visible changes and affirm the patient’s commitment to their behaviour modification • **Build Self-Efficacy**: Strengthen the patient’s belief in their ability to maintain the changes by highlighting their successes and reinforcing their autonomy • **Celebrate Achievements**: Celebrate milestones and reinforce that their efforts are valid and important for long-term success
Maintenance	Patient has maintained behaviour change for six months or more	• Review and revise action plan so that strategies are geared towards maintaining behaviour change and/or the goal health state achieved (e.g. healthy weight, incorporating vegetables into at least one meal a day, performing resistance training two times a week) • If possible, schedule time to monitor and review every 6–12 months	• **Sustain Change**: Help patients consolidate gains and maintain changes to prevent regression, recognising that this stage can last from months to a lifetime • **Prevent regression:** Identify potential triggers such as stress and social events and develop management strategies. Acknowledge that it may occur, viewing it as a learning opportunity rather than failure. Guide patients through"regression crises"to rebuild self-efficacy and motivation • **Learn from Setbacks**: When regression occurs, support the patient in reassessing their plan, learning from the experience, and reinitiating the change process

## Conclusions

In summary, obesity has detrimental effects on physical function and independence and weight loss strategies that fail to adequately consider diet composition and exercise may further impair musculoskeletal health. Sustainable weight loss requires a personalised approach that prioritises sufficient protein intake, calcium, vitamin D and other nutrients essential for muscle and bone preservation. Adherence to weight loss strategies is equally critical, with the most effective approaches being those that are easy for individuals to sustain long-term. Similarly, exercise programmes must be tailored and progressive, with RT serving as the cornerstone of exercise prescribed in the context of weight loss. To optimise outcomes, clinicians should prioritise making RT both accessible and enjoyable, particularly for vulnerable populations such as older adults.

The practical diet and exercise recommendations provided in this review can serve as a useful guide for those contemplating, commencing or prescribing weight loss interventions, but it should be recognised that behavioural change is integral to the success of any weight loss programme. By assessing an individual’s readiness to change and tailoring interventions accordingly, clinicians can enhance adherence, promote lasting change and maximise health outcomes.

## Data Availability

No datasets were generated or analysed during the current study.
